# SARS-CoV-2 lineage B.1.1.7 is associated with greater disease severity among hospitalised women but not men: multicentre cohort study

**DOI:** 10.1136/bmjresp-2021-001029

**Published:** 2021-09-20

**Authors:** Oliver Stirrup, Florencia Boshier, Cristina Venturini, José Afonso Guerra-Assunção, Adela Alcolea-Medina, Angela Beckett, Themoula Charalampous, Ana da Silva Filipe, Sharon Glaysher, Tabassum Khan, Raghavendran Kulasegaran Shylini, Beatrix Kele, Irene Monahan, Guy Mollett, Matthew Parker, Emanuela Pelosi, Paul Randell, Sunando Roy, Joshua Taylor, Sophie Weller, Eleri Wilson-Davies, Phillip Wade, Rachel Williams, The COG-UK-HOCI Variant substudy consortium, The COVID-19 Genomics UK (COG-UK) consortium, Andrew Copas, Maria-Teresa Cutino-Moguel, Nick Freemantle, Andrew C Hayward, Alison Holmes, Joseph Hughes, Tabitha Mahungu, Gaia Nebbia, David Partridge, Cassie Pope, James Price, Samuel Robson, Kordo Saeed, Thushan de Silva, Luke Snell, Emma Thomson, Adam A Witney, Judith Breuer

**Affiliations:** 1 Institute for Global Health, University College London, London, UK; 2 Department of Infection, Immunity and Inflammation, UCL Great Ormond Street Institute of Child Health, University College London, London, UK; 3 Department of Genetics & Genomic Medicine, UCL Great Ormond Street Institute of Child Health, University College London, London, UK; 4 Centre for Clinical Infection and Diagnostics Research, School of Immunology and Microbial Sciences, King’s College London, London, UK; 5 Infection Sciences, Viapath, London, UK; 6 Centre for Enzyme Innovation, University of Portsmouth, Portsmouth, UK; 7 School of Biological Sciences, University of Portsmouth, Portsmouth, UK; 8 MRC-University of Glasgow Centre for Virus Research, Glasgow, UK; 9 Portsmouth Hospitals University NHS Trust, Queen Alexandra Hospital, Portsmouth, UK; 10 Division of Infection, The Royal London Hospital, Barts Health NHS Trust, London, UK; 11 Institute for Infection and Immunity, St George’s University of London, London, UK; 12 Sheffield Bioinformatics Core, The University of Sheffield, Sheffield, UK; 13 Sheffield Institute for Translational Neuroscience, The University of Sheffield, Sheffield, UK; 14 Sheffield Biomedical Research Centre, The University of Sheffield, Sheffield, UK; 15 Southampton Specialist Virology Centre, University Hospital Southampton NHS Foundation Trust, Southampton, UK; 16 Department of Infection and Immunity, North West London Pathology, London, UK; 17 Department of Microbiology, South West London Pathology, St. George’s Hospital, London, UK; 18 Department of Virology, Royal Free London NHS Foundation Trust, London, UK; 19 Sheffield Teaching Hospitals NHS Foundation Trust, Sheffield, UK; 20 The Florey Institute for Host-Pathogen Interactions & Department of Infection, Immunity and Cardiovascular Disease, Medical School, University of Sheffield, Sheffield, UK; 21 Institute of Clinical Trials and Methodology, University College London, London, UK; 22 Institute of Epidemiology and Health Care, University College London, London, UK; 23 Department of Infectious Disease, Faculty of Medicine, Imperial College London, London, UK; 24 Hammersmith Hospital, Imperial College Healthcare NHS Trust, London, UK; 25 Department of Infectious Diseases, Guy’s and St Thomas’ Hospital NHS Foundation Trust, London, UK; 26 Infection Care Group, St George’s University Hospitals NHS Foundation Trust, London, UK; 27 Imperial College Healthcare NHS Trust, London, UK; 28 School of Pharmacy and Biomedical Sciences, University of Portsmouth, Portsmouth, UK; 29 Microbiology Innovation and Research Unit (MIRU), Department of Microbiology, University Hospital Southampton NHS Foundation Trust, Southampton, UK; 30 Faculty of Medicine, Clinical and Experimental Sciences, University of Southampton, Southampton, UK; 31 Department of Microbiology, Great Ormond Street Hospital for Children NHS Foundation Trust, London, UK

**Keywords:** COVID-19, viral infection

## Abstract

**Background:**

SARS-CoV-2 lineage B.1.1.7 has been associated with an increased rate of transmission and disease severity among subjects testing positive in the community. Its impact on hospitalised patients is less well documented.

**Methods:**

We collected viral sequences and clinical data of patients admitted with SARS-CoV-2 and hospital-onset COVID-19 infections (HOCIs), sampled 16 November 2020 to 10 January 2021, from eight hospitals participating in the COG-UK-HOCI study. Associations between the variant and the outcomes of all-cause mortality and intensive therapy unit (ITU) admission were evaluated using mixed effects Cox models adjusted by age, sex, comorbidities, care home residence, pregnancy and ethnicity.

**Findings:**

Sequences were obtained from 2341 inpatients (HOCI cases=786) and analysis of clinical outcomes was carried out in 2147 inpatients with all data available. The HR for mortality of B.1.1.7 compared with other lineages was 1.01 (95% CI 0.79 to 1.28, p=0.94) and for ITU admission was 1.01 (95% CI 0.75 to 1.37, p=0.96). Analysis of sex-specific effects of B.1.1.7 identified increased risk of mortality (HR 1.30, 95% CI 0.95 to 1.78, p=0.096) and ITU admission (HR 1.82, 95% CI 1.15 to 2.90, p=0.011) in females infected with the variant but not males (mortality HR 0.82, 95% CI 0.61 to 1.10, p=0.177; ITU HR 0.74, 95% CI 0.52 to 1.04, p=0.086).

**Interpretation:**

In common with smaller studies of patients hospitalised with SARS-CoV-2, we did not find an overall increase in mortality or ITU admission associated with B.1.1.7 compared with other lineages. However, women with B.1.1.7 may be at an increased risk of admission to intensive care and at modestly increased risk of mortality.

**Key messages**

- In a multicentre cohort we did not find an overall increase in mortality or intensive therapy unit admission associated with B.1.1.7 among hospitalised patients, but women with B.1.1.7 may be at an increased risk of admission to intensive care and at modestly increased risk of mortality.

- The impact of SARS-CoV-2 lineage B.1.1.7 on disease severity appears to be dependent on the patient’s sex, meaning that its health impacts and healthcare burden will differ from earlier lineages of the virus and that monitoring of disease course by sex should be considered for other viral lineages with increased transmissibility.

## Introduction

The emergence of SARS-CoV-2 lineage B.1.1.7 in South East England has been found to be associated with an estimated 70% increased rate of community transmission compared with previously circulating variants.^1–3^ Lineage B.1.1.7 became the dominant lineage in the UK in winter 2020/2021. It has also been detected in over 120 countries outside the UK,^4^ and was assigned the label of variant of concern Alpha by WHO.^5^

Lineage B.1.1.7 has acquired an unusually large number of mutations and deletions in a short period of time^1–3^; specifically 14 non-synonymous single nucleotide polymorphisms and 3 amino acid deletions, with 8 of these 17 amino acid changes occurring in the spike protein, responsible for receptor binding and a major immunogenic target. At least three of the spike protein changes are associated with in vitro biological changes. A tyrosine substitution at position 501 in the spike protein receptor binding domain has been shown to increase binding to the ACE2 receptor, while deletion of spike protein amino acids 69/70 reduces antibody neutralisation by convalescent sera.^6 7^ The potential that so many mutations might change B.1.1.7 virulence has been examined epidemiologically using data largely from community-collected samples.^8–11^ However, there are few data on the impact of B.1.1.7 infection as compared with other variants on disease outcomes in hospitalised patients.

We investigated the potential associations between the B.1.1.7 variant and the outcomes of mortality and intensive therapy unit (ITU) admission both in patients admitted with COVID-19 and hospital onset COVID-19 infections (HOCIs) in the COG-UK-HOCI study. The main objective was to estimate the overall effect of the variant on each of these outcomes, and we also evaluated whether the impact of the variant differed according to patient characteristics.

## Methods

### Sequence and patient meta-data

Data were collected from five NHS hospitals within London and three outside. The first SARS-CoV-2-positive sample from all inpatients tested through hospital laboratories between 16 November 2020 and 10 January 2021 was sequenced. In addition, metadata were collected from clinical records on patient age, sex (as binary M/F), comorbidities as identified by the COVID-19 Greenbook^12^ (including obesity with body mass index ≥35 kg/m^2^), care home residence, pregnancy, ethnicity, date of hospital admission, ward location and first SARS-CoV-2-positive test for all samples plus dates of admission to the ITU and all-cause death where these events occurred.

Inpatients were classified as those admitted with SARS-CoV-2 plus cases which were identified after admission, with the latter termed HOCI cases and subdivided into indeterminate healthcare-associated infections (HCAIs) diagnosed 3–7 days after admission and probable/definite HCAIs diagnosed ≥8 days postadmission.^13^ The primary outcomes for analysis were the events of death and of ITU admission. Events were included in the analysis within 28 days of hospital admission for those admitted with COVID-19 and within 28 days of diagnosis for HOCI cases.

### SARS-CoV-2 sequencing

Samples were sequenced by Nanopore or Illumina methods as part of the COVID-19 Genomics UK Consortium (COG-UK). To maximise success, 4/8 labs sequenced only those samples with quantitative PCR cycle thresholds (ct) values of ≤32 or equivalent. Sequences were assigned to lineages using COG-UK Pangolin.^14^

### Statistical analysis

Only patients with admission to hospital and HOCIs were included in the statistical analysis of the clinical outcomes of mortality and ITU admission. Mortality and ITU admission were modelled as time-to-event outcomes, from time of hospital admission for those admitted with COVID-19 and from time of diagnosis for HOCI cases, censored at 28 days. Analyses of ITU admission were also censored at patient death. Both outcomes were censored at date of data collection for these variables for each site (between 15 January and 22 February 2021). Mixed effects Cox models were used with adjustment for sex, patient age (using 5-knot restricted cubic spline), number of comorbidities (none, one, two, ≥three), care home residence, pregnancy, ethnicity (white, black, Asian, mixed or other) and sample week with separate parameters for London sites and for other sites grouped using the R package coxme V.2.2–16.^15^ A 5-knot restricted cubic spline^16^ was used for patient age in all analyses to allow flexibility in modelling the relationship with each outcome while maintaining a consistent model structure. Random intercept terms were included to reflect clustering of outcomes within hospital and weekly periods nested within hospitals. Cox models were stratified by HOCI status (allowing for different baseline hazard functions in patients admitted with COVID-19 vs HOCI groups).

Outcomes were analysed on a complete case basis with regard to patient characteristics. This decision was based on the availability of complete data for >90% of patients and the fact that Cox regression gives asymptotically unbiased estimates of an association of interest as long as the missingness is not dependent on both outcome (ie, death or ITU admission) and exposure (B.1.1.7 status).^17 18^ The variable of obesity was analysed as ‘morbid obesity’ versus ‘no record of morbid obesity’ on examination of case notes, and was included in statistical models within the ordinal comorbidities variable.

The primary aim of the analysis was to estimate the overall association between the B.1.1.7 vs non-B.1.1.7 strain and the risk of each of the outcomes considered. Exploratory secondary analyses also evaluated interactions between B.1.1.7 status and patient characteristics in estimating the effect on each outcome. Analyses were conducted in R V.4.0.2, using tidyverse collection of packages with all plots generated using ggplot2 and survminer.^19–22^

### Patient and public involvement

This was an analysis of retrospectively collected data, for which the planning, data collection and analysis were all carried out during a period of extreme pressure on the UK health service due to the COVID-19 epidemic. As such, there was no patient and public engagement in the conduct of this research and it would not be possible to disseminate the findings directly to participants.

## Results

### Study dataset

Between 16 November 2020 and 10 January 2021, SARS-CoV-2 RNA-positive upper respiratory tract samples from 2341 inpatients were sequenced from the eight participating sites (table 1 and online supplemental figure S1). Analysis of clinical outcomes was carried out in 2147 inpatients with all data available. The prevalence of lineage B.1.1.7 was highest in London and Hampshire (South of England), but substantially increased at all sites over the study period (online supplemental figure S2).

10.1136/bmjresp-2021-001029.supp1 Supplementary data

**Table 1:** Proportion of SARS-CoV-2 due to lineage B.1.1.7 for all inpatient sequenced samples according to patient characteristics

	Lineage B.1.1.7 (n=1107)	Not lineage B.1.1.7 (n=1234)	Total (n=2341)
Age group (years)			
0–11	15 (62.5)	9 (37.5)	24 (100)
12–24	20 (58.8)	14 (41.2)	34 (100)
25–34	61 (62.9)	36 (37.1)	97 (100)
35–49	159 (56.2)	124 (43.8)	283 (100)
50–69	371 (53.2)	326 (46.8)	697 (100)
70–79	208 (42.2)	285 (57.8)	493 (100)
80+	273 (38.3)	440 (61.7)	713 (100)
Sex			
Female	534 (46.1)	624 (53.9)	1158 (100)
Male	573 (48.4)	610 (51.6)	1183 (100)
Sample week starting			
16 November 2020	15 (8.4)	164 (91.6)	179 (100)
23 November 2020	26 (11.6)	198 (88.4)	224 (100)
30 November 2020	59 (24.2)	185 (75.8)	244 (100)
07 December 2020	55 (26.8)	150 (73.2)	205 (100)
14 December 2020	138 (43.8)	177 (56.2)	315 (100)
21 December 2020	220 (54.6)	183 (45.4)	403 (100)
28 December 2020	361 (75.2)	119 (24.8)	480 (100)
04 January 2021	233 (80.1)	58 (19.9)	291 (100)
Patient class			
HCW	7 (36.8)	12 (63.2)	19 (100)
CAI*	847 (55.1)	689 (44.9)	1536 (100)
Indeterminate HCAI†	54 (25.4)	159 (74.6)	213 (100)
Probable/definite HCAI‡	199 (34.7)	374 (65.3)	573 (100)
Region			
Glasgow	91 (31.6)	197 (68.4)	288 (100)
Hampshire	74 (60.2)	49 (39.8)	123 (100)
London	871 (65.7)	455 (34.3)	1326 (100)
South Yorkshire	71 (11.8)	533 (88.2)	604 (100)
Ethnicity			
White	540 (39.4)	829 (60.6)	1369 (100)
Black	174 (53.4)	152 (46.6)	326 (100)
Asian	118 (63.1)	69 (36.9)	187 (100)
Mixed or other	186 (67.1)	91 (32.9)	277 (100)
Unknown	89 (48.9)	93 (51.1)	182 (100)
Patient characteristics			
Obese (BMI ≥35)	122 (51) (N=1107)	117 (49) (N=1234)	239 (100) (N=2341)
Pregnant	25 (55.6) (N=1103)	20 (44.4) (N=1234)	45 (100) (N=2337)
Care home resident	45 (36.3) (N=1107)	79 (63.7) (N=1232)	124 (100) (N=2339)
Comorbidities			
None	337 (56.6)	258 (43.4)	595 (100)
One	307 (47.3)	342 (52.7)	649 (100)
Two	261 (45.9)	308 (54.1)	569 (100)
Three or more	202 (38.4)	324 (61.6)	526 (100)
Not recorded	0 (0)	2 (100)	2 (100)
Died within 28 days	217 (41.2) (N=1106)	310 (58.8) (N=1230)	527 (100) (N=2336)
Admitted to ITU within 28 days§	220 (65.3) (N=1081)	117 (34.7) (N=1214)	337 (100) (N=2295)

Data shown as *n* (%), with (N) with available data shown where missing values possible.

*Diagnosed at or ≤2 days from admission.

†Diagnosed 3–7 days from admission.

‡Diagnosed ≥8 days from admission.

§Excluding patients admitted to ITU prior to SARS-CoV-2 diagnosis.

BMI, body mass index; CAI, community-acquired infection; HCAI, healthcare-associated infection; HCW, healthcare worker; ITU, intensive therapy unit.

### Mortality outcome

**Figure 1:**
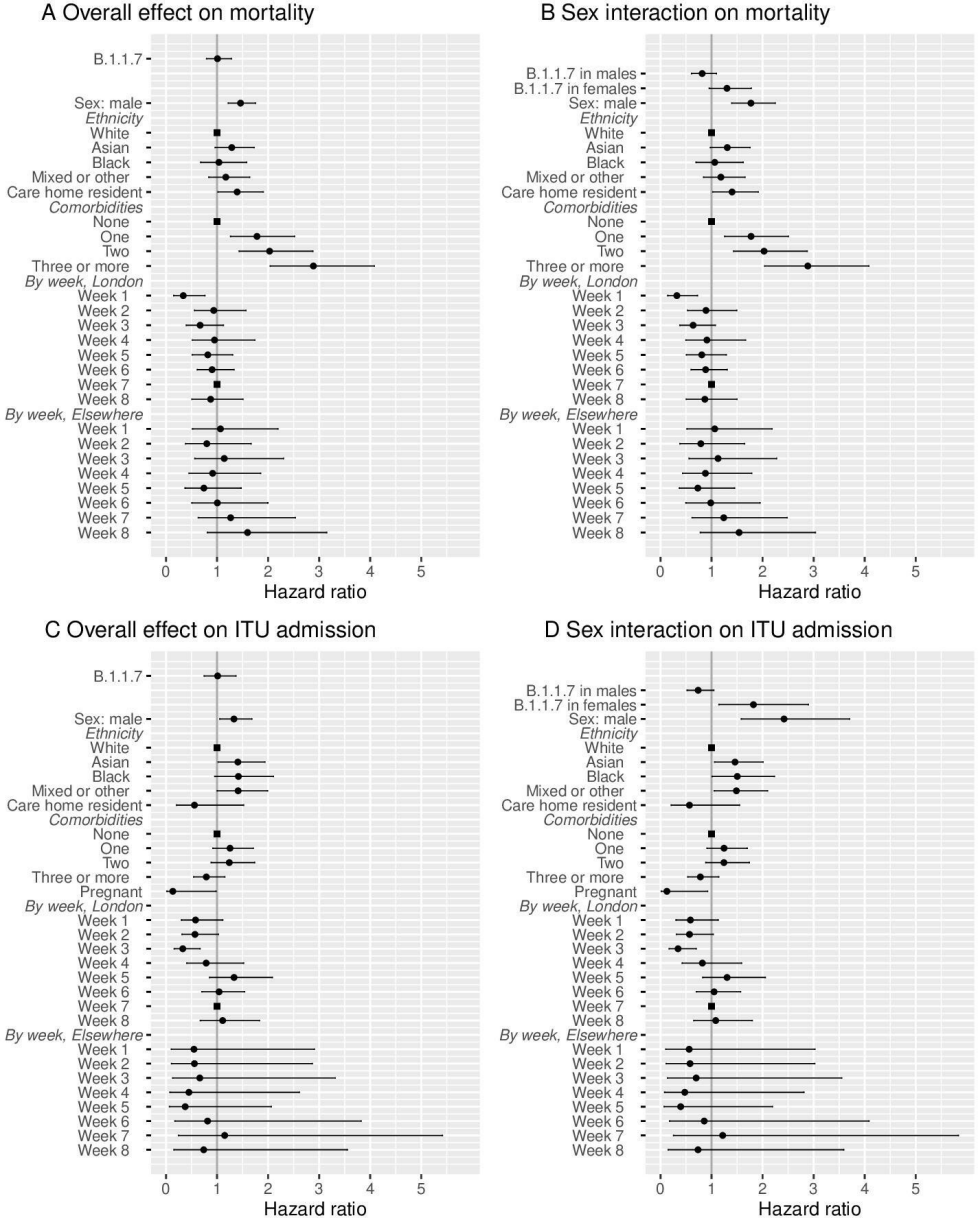
Results of mixed effect Cox regression models for death and intensive therapy unit (ITU) admission, shown as HR (●, ■ for reference categories) with 95% CI. Results are displayed for outcomes of mortality (A, B) and ITU admission (C, D), both for the overall effect of B.1.1.7 variant (A, C) and with sex-specific effects of B.1.1.7 (B, D). Models were all also adjusted by age using natural cubic splines (as shown in figure 2).

**Figure 2:**
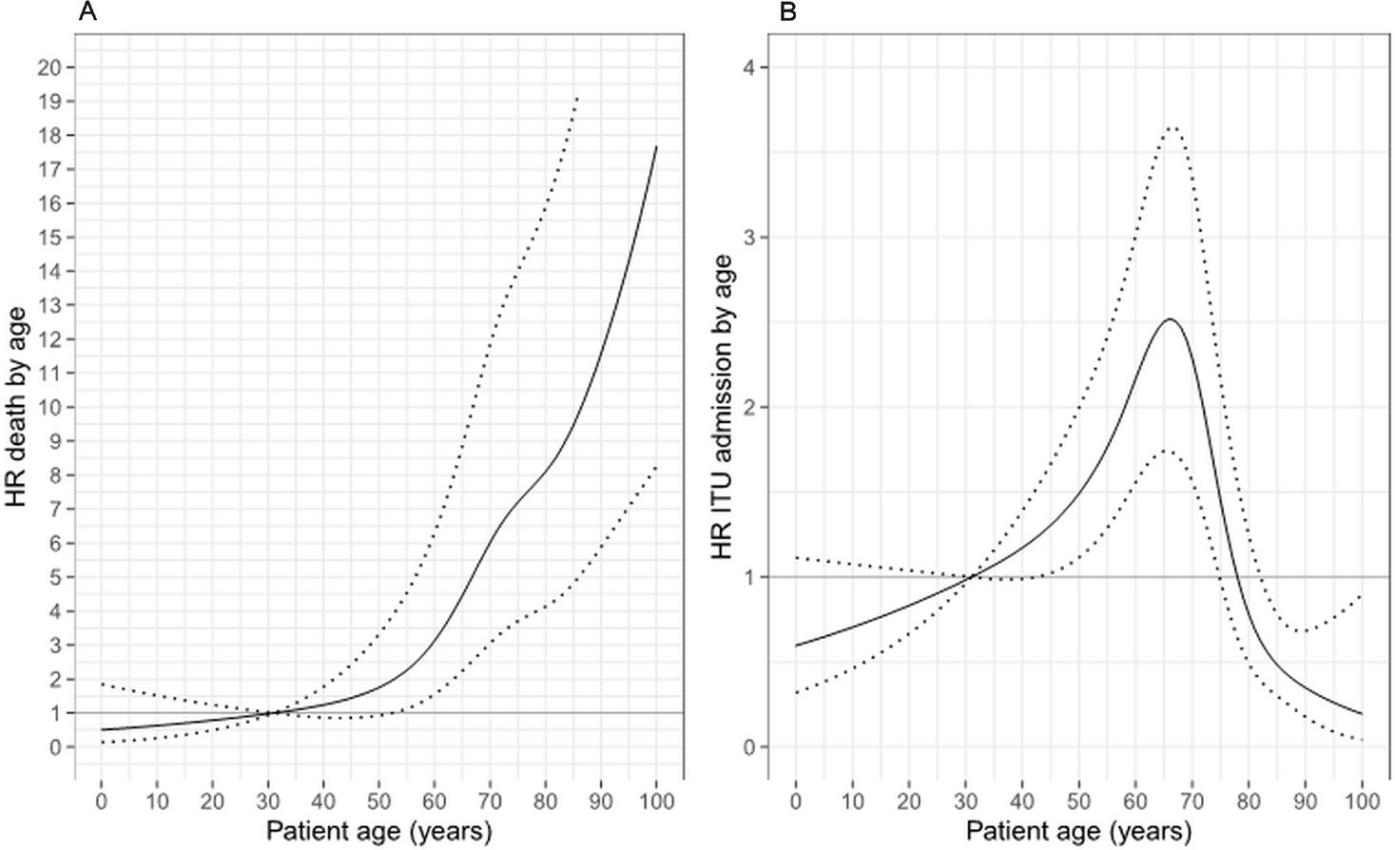
Plots of estimated HR for (A) death and (B) intensive therapy unit (ITU) admission in relation to age for mixed effects Cox regression models with single overall effect of B.1.1.7 variant (95% CIs shown as dotted lines). Following from the parameterisation of the model, HRs are shown relative to hazard at age of 31 years.

Death within 28 days was reported in 527 (22.5%) of the 2341 patients. Death was recorded as having occurred following discharge with date of death missing in five, and these patients have been excluded from analyses. Death within 28 days was recorded in 494/2147 (23.0%) of the patients with all data available, with full 28 days of follow-up in 939/1653 (56.8%) of the other patients. On mixed effects multivariable Cox regression, the overall HR for mortality of lineage B.1.1.7 was 1.01 (95% CI 0.79 to 1.28, p=0.94) (figure 1, online supplemental table S1). Male sex was found to be a substantial risk factor for mortality (HR 1.46 vs female, 95% CI 1.22 to 1.75; p<0.001) and age was also strongly associated with the risk of death (figure 2). The risk of death was higher in care home residents (HR 1.39, 95% CI 1.02 to 1.90, p=0.04) and those with one or more significant comorbidities (HR 1.78 (95% CI 1.26 to 2.52) for one comorbidity, 2.03 (95% CI 1.43 to 2.88) for two and 2.89 (95% CI 2.04 to 4.08) for ≥three versus none; p<0.001). Those with ethnicity other than white were estimated to be at higher risk of death, but ethnicity was not a statistically significant predictor when evaluated over all categories (p=0.36). No pregnant women died and so this variable was dropped from the model as a perfect predictor.

The addition of an interaction term between B.1.1.7 status and patient sex for the effect on mortality led to an improvement in model fit (p=0.01 interaction test, p=0.04 lineage B.1.1.7 effects by sex vs no B.1.1.7 effect, on likelihood ratio tests (LRT)). The estimated HR for mortality of lineage B.1.1.7 vs non-B.1.1.7 was 0.82 (95% CI 0.61 to 1.10, p=0.177) in male patients and 1.30 (95% CI 0.95 to 1.78, p=0.096) in female patients. No improvement to model fit was provided by the addition of an interaction between B.1.1.7 status and patient age (p=0.48, LRT with 4 df), ethnicity (p=0.67, LRT with 3 df) or comorbidity category (p=0.33, LRT with 3 df).

A statistically significant interaction was found between the effect of B.1.1.7 and care home residence (p=0.03, LRT with 1 df), with those care home residents with B.1.1.7 infection estimated to be at lower risk of death (HR 0.52, 95% CI 0.27 to 1.02, p=0.057) with a non-significant increase in the risk for death associated with B.1.1.7 for non-care home residents (1.09, 95% CI 0.85 to 1.41, p=0.49). We attempted to fit a model including interaction on both sex and care home residence status, but convergence of parameter estimates failed. The model with interaction on sex had the lowest Akaike information criterion of all fitted models and, given also the relatively small number of care home residents in the dataset, we therefore focus on this model for interpretation and analysis.

**Figure 3:**
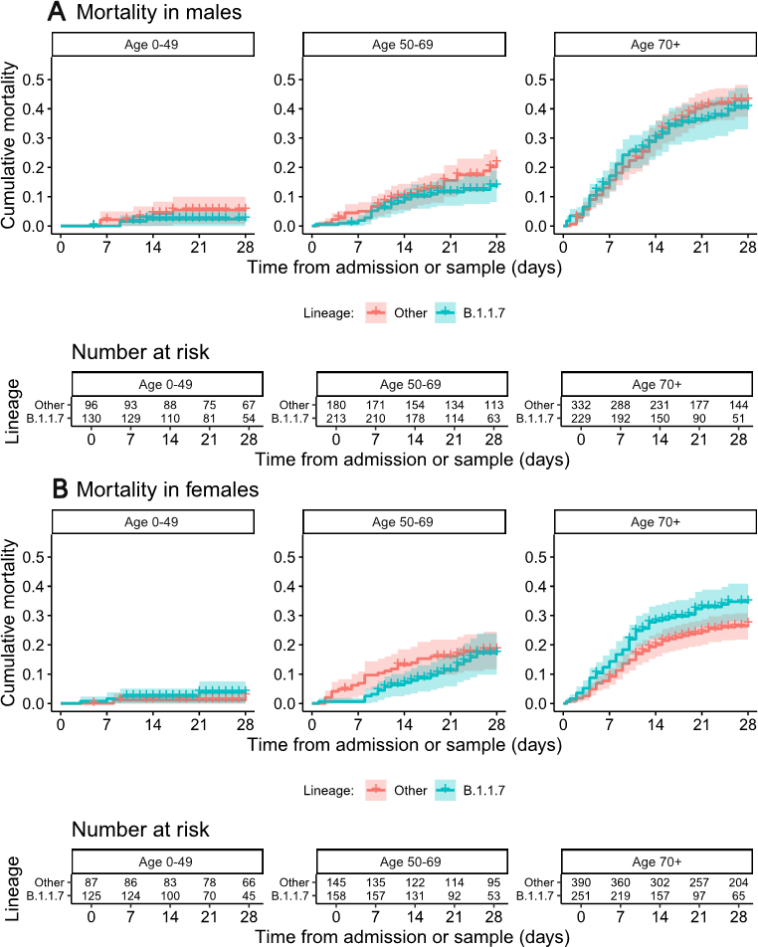
Kaplan-Meier plots of all-cause mortality among all inpatients in relation to lineage B.1.1.7 status, plotted according to patient sex and age categories. Date of sampling is used as the ‘zero’ time point for hospital-onset COVID-19 infections, with date of admission used for other patients. Naïve 95% CIs are plotted for illustrative purposes (these are not derived from the multilevel Cox models described).

Kaplan-Meier plots of mortality in relation to B.1.1.7 status are presented according to patient sex and age categories in figure 3 (also provided separately for non-HOCI and HOCI inpatients in online supplemental figures S3 and S4, with HR estimates in online supplemental table S2).

### ITU admission outcome

Admission to ITU within 28 days was reported in 337 (14.4%) of 2341 inpatients (excluding 46 HOCI cases diagnosed after admission to ITU). On mixed effects multivariable Cox regression, the overall HR for ITU admission for lineage B.1.1.7 was 1.01 (95% CI 0.75 to 1.37, p=0.94) (figure 1, online supplemental table S1). Within this model, male sex was a substantial risk factor for ITU admission (HR 1.33, 95% CI 1.05 to 1.68; p=0.02). Age was also strongly associated with the risk of ITU admission, although the relationship estimated was non-linear with the greatest risk of this outcome at 65 years of age (figure 2). The risk of ITU admission was higher in those with one or two significant comorbidities (HR 1.25 (95% CI 0.92 to 1.71) for one comorbidity, 1.24 (95% CI 0.89 to 1.74) for two and 0.79 (95% CI 0.54 to 1.15) for ≥three versus none; p=0.03). Those with ethnicity other than white were estimated to be at higher risk of ITU admission, but ethnicity was not a statistically significant predictor evaluated over all categories (p=0.09). Pregnant women were found to be at lower risk of ITU admission (HR 0.13, 95% CI 0.02 to 0.98, p=0.048).

**Figure 4:**
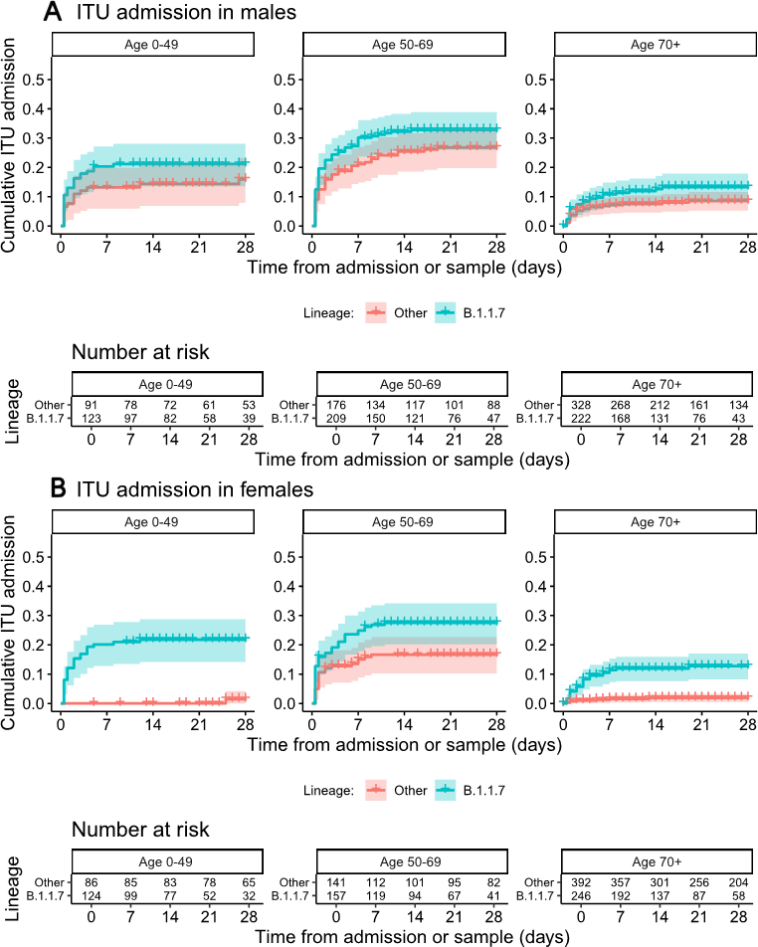
Kaplan-Meier plots of intensive therapy unit (ITU) admission among all inpatients in relation to lineage B.1.1.7 status, plotted according to patient sex and age categories. Date of sampling is used as the ‘zero’ time point for hospital-onset COVID-19 infections, with date of admission used for other patients. Naïve 95% CIs are plotted for illustrative purposes (these are not derived from the multilevel Cox models described).

The addition of an interaction term between B.1.1.7 status and patient sex for the effect on ITU admission led to an improvement in model fit (p=0.0004 interaction test, p=0.002 lineage B.1.1.7 effects by sex vs no B.1.1.7 effect, LRTs). The estimated HR for ITU admission for lineage B.1.1.7 vs non-B.1.1.7 was 0.74 (95% CI 0.52 to 1.04, p=0.086) in male patients and 1.82 (95% CI 1.15 to 2.90, p=0.011) in female patients. There was no evidence for an interaction of B.1.1.7 status with patient age (p=0.11, LRT with 4 df), ethnicity (p=0.74, LRT with 3 df), comorbidity category (p=0.79, LRT with 3 df), pregnancy (p=0.42, LRT with 1 df) or care home residence (p=0.24, LRT with 1 df) with ITU admission as the outcome. Kaplan-Meier plots of ITU admission in relation to B.1.1.7 status are presented according to patient sex and age categories in figure 4 (also provided separately for non-HOCI and HOCI inpatients in online supplemental figures S5 and S6, with HR estimates in online supplemental table S2).

## Discussion

Our findings provide the largest dataset on disease severity in hospitalised patients with lineage B.1.1.7 and the only one based on routine sequencing of all specimens from multiple hospitals. The overall hazard of mortality and ITU were unchanged for patients with lineage B.1.1.7 in comparison to other viral variants (HR 1.01, 95% CI 0.79 to 1.28 and HR 1.01, 95% CI 0.75 to 1.37, respectively). These findings are in line with the results of a much smaller analysis of 341 (n=198 with B.1.1.7) hospital inpatients with viral sequencing over a similar time period in London, which found an adjusted mortality risk ratio for B.1.1.7 of 1.02 (95% CI 0.76 to 1.38).^23^ However, in contrast with this smaller study we also found evidence that B.1.1.7 infection appears to have a different impact on the disease course according to sex among hospitalised patients with SARS-CoV-2 infection, with increased hazard of both mortality and ITU admission associated with the variant for female but not male patients.

Several larger studies of disease severity in the UK have used PCR Spike (S) gene target failure (SGTF) as a surrogate for lineage B.1.1.7.^8–11^ These studies, based on community testing data, all found evidence of an overall increased risk of mortality associated with lineage B.1.1.7, with reported HRs of 1.64 (95% CI 1.32 to 2.04) by Challen *et al*,^8^ 1.55 (95% CI 1.39 to 1.72) by Davies *et al*,^9^ 1.67 (95% CI 1.34 to 2.09) by Grint *et al*^10^ and 1.59 (95% CI 1.25 to 2.03) by Patone *et al*.^11^ In the UK, SGTF is only available as a marker for a subset of those patients who were first positive for SARS-CoV-2 on testing within the community; most people who die of COVID-19 were not previously tested within the community^8^ and the relevant PCR assay is not used by all laboratories, meaning that SGTF status is only available for 8.6% of deaths.^9^ SGTF is an imperfect predictor of lineage B.1.1.7, and is much less accurate as a marker when prevalence of the variant is low (before mid-November 2020 in the UK).^24^

The apparent overall differences in mortality risk observed in the SGTF analyses in comparison to our study do not necessarily represent inconsistent findings. Studies that are limited to patients who test positive in the community may be subject to selection biases linked to propensity to present for testing or rapidity of disease progression, while analyses that include only data from inpatients will not reflect the characteristics of the population as a whole. For example, increased disease severity may result in a higher proportion of subjects reaching the threshold for admission to hospital but not affect the mortality rate among those who are admitted to hospital. Our study also includes a subset of patients with probable nosocomial infection, whose characteristics and comorbidity profile differs greatly from the UK population as whole.^25^

Individuals testing positive in the community for an SGTF-associated variant had higher risk of hospitalisation, with OR of 1.58 (95% CI 1.50 to 1.67).^26^ This result was confirmed by a study of national health register-data from Denmark including 18 499 patients with viral genomes available in the period 1 January to 9 February 2021 which found an adjusted OR of 1.64 (95% CI 1.32 to 2.04) for hospitalisation for B.1.1.7 compared with other lineages.^27^ Taken together with the findings regarding mortality in the UK,^8–11^ these results are consistent with an increased risk of mortality and hospitalisation among patients testing positive for B.1.1.7 in the community but no overall increase in mortality among the subset of patients admitted to hospital.

We found a significantly increased risk of both mortality (30%) and ITU admission (82%) in hospitalised female patients infected with B.1.1.7 but not in male patients. In contrast, studies of community-tested individuals found no interaction with sex for the effect of B.1.1.7 on mortality,^9 11^ critical care admission^11^ or risk of hospitalisation.^26^ However, these studies were all conducted among patients who first tested positive for SARS-CoV-2 within the community, and therefore they cannot rule out an interaction with sex for the impact of B.1.1.7 on disease severity among all people infected with the virus or among all those admitted to hospital. Nationally collated data show that females accounted for 33.2% of patients admitted to ITU with COVID-19 in London, East and South East England between 1 September and 30 November 2020 rising to 36.2%, between 1 December 2020 and 21 January 2021 when lineage B.1.1.7 predominated.^28^

There is evidence that the total number of pregnant women requiring intensive care in the UK was higher in late 2020/early 2021 in comparison to the first wave of the COVID-19 epidemic in Spring 2020,^29^ and that the proportion of symptomatic pregnant women requiring admission to ITU increased as lineage B.1.1.7 became dominant.^30^ This would be consistent with a differential impact of the B.1.1.7 variant on women in comparison to men. However, we should also note that our analyses found pregnancy itself to be negatively associated with ITU admission, conditional on age, sex, viral variant, comorbidities and other patient characteristics. This could possibly be due to unobserved or residual confounding not fully captured by our recording and analysis of patient characteristics.

An impact of lineage B.1.1.7 on females that is not observed in males could potentially be explained by physiological differences. For example, increased ACE2 expression in females has been posited as one explanation for the relatively lower mortality and morbidity observed for COVID-19 for women in comparison to men.^31 32^ Lineage B.1.1.7 has mutations that increase binding of the viral spike protein to ACE2, thereby providing a plausible mechanism by which the new variant might have a differential effect on disease severity in males and females.^6 31 33^ Our results suggest a reduction in the risk of mortality or ITU admission associated with B.1.1.7 in comparison to other viral lineages among male inpatients, although this finding was not definitive with HR 95% CIs that included no effect for both outcomes. However, this is against a backdrop of the established increased risk for male versus female patients for pre-B.1.1.7 strains.^34^ Our results indicate an approximate equalisation of risk for otherwise equivalent male and female inpatients with B.1.1.7 infection.

Although ours is substantially the largest study of hospitalised patients with confirmed lineage B.1.1.7 and non-B.1.1.7 SARS-Cov-2 infection, it has a number of limitations. Primarily, while evaluation of disease severity among only hospital inpatients can give useful information on disease course and progression, analysis of only these patients cannot provide information on disease severity across all SARS-CoV-2 infections in the population as a whole. In addition, ITU admission can be difficult to interpret as a measure of disease severity among inpatients. For instance, admission to ITU may reflect the presence of severe disease and local decisions around the benefit or lack thereof to frail patients, which may be influenced by bed numbers and availability of respiratory support in non-critical care settings. Our primary analysis also includes cases of hospital-acquired infection but exclusion of these HOCI cases from our analyses yielded similar findings (online supplemental table S2).

A further limitation of our analysis is that we do not have any information on vaccination status for individual patients. Our dataset covers a period in which a national vaccination programme was being initiated for HCWs and the elderly population in the UK, starting with those aged 80 years and above from 8 December 2020. This is a potential explanation for the observed protective interaction effect between care home residence and B.1.1.7 on mortality, as care home residents were prioritised for vaccination around the time that this viral variant was increasing in prevalence. Vaccine breakthrough infections are well described, particularly in partially vaccinated subjects.^35^

Our analysis was focused on the effect of lineage B.1.1.7 as a single exposure of interest for each outcome variable. However, we also considered 5 potential interactions with viral lineage for the outcome of mortality and 6 for ITU admission, giving a total of 13 hypothesis tests of interest. A conservative use of the Bonferroni correction gives a p value cut-off adjusted to 0.0038 from the commonly used threshold of 0.05, using which we would consider there to be robust statistical evidence for an interaction with sex on the outcome of ITU admission but not for the interactions between sex or care home status on mortality.

### Implications

Although lineage B.1.1.7 was not associated with an overall increase in mortality among hospitalised patients, our investigation suggests that lineage B.1.1.7 may be associated with higher ITU admission and death in females compared with non-B.1.1.7 within this group. The dominance of lineage B.1.1.7 in the UK precluded further comparison with earlier non-B.1.1.7 variants, and there is now concern regarding the spread of other lineages in the UK and elsewhere.^36^ There is a need for ongoing large-scale sequencing of SARS-CoV-2 cases linked to data on patient characteristics and outcomes in order to generate timely information regarding the associations between viral lineages and disease severity. Monitoring of disease course by sex should be considered for other viral lineages with increased transmissibility.

## Data Availability

The sequence data analysed are included within publicly available datasets (https://www.cogconsortium.uk/data/). However, due to data governance restrictions it is not possible to share the associated patient characteristics and clinical outcome data for the analysis described, as these are considered sensitive and full anonymisation is not possible. The corresponding author (OTS) affirms that the manuscript is an honest, accurate and transparent account of the study being reported; that no important aspects of the study have been omitted and that any discrepancies from the study as planned have been explained.
