# Supplementary material for: SARS-CoV-2 lineage B.1.1.7 is associated with greater disease severity among hospitalised women but not men: multicentre cohort study
Source: BMJ Open Respir Res. 2021 Sep 20;8(1):e001029. doi: 10.1136/bmjresp-2021-001029 (PMC8453594; doi:10.1136/bmjresp-2021-001029)

Supplementary Table 1 Results of mixed effect Cox regression models for death and intensive therapy unit admission, shown as hazard ratio (95% CI) [P-value]. Models were also adjusted by age using natural cubic splines (as shown in Figure 2). A graphical plot of these results is provided in Figure 1 of the main text.

	**Outcome: death**		**Outcome: ITU admission**	
**variable**	**Overall effect**	**Interaction with sex**	**Overall effect**	**Interaction with sex**
**Var.: B.1.1.7**	1.01 (0.79 to 1.28) [0.943]		1.01 (0.75 to 1.37) [0.939]	
**Var.: B.1.1.7 in males**		0.82 (0.61 to 1.1) [0.177]		0.74 (0.52 to 1.04) [0.086]
**Var.: B.1.1.7 in females**		1.3 (0.95 to 1.78) [0.096]		1.82 (1.15 to 2.9) [0.011]
**Sex: male**	1.46 (1.22 to 1.75) [P<0.001]	1.77 (1.4 to 2.25) [0]	1.33 (1.05 to 1.68) [0.017]	2.42 (1.58 to 3.71) [0]
**Ethnicity**	[0.364]	[0.316]	[0.088]	[0.038]
**White**	1.0 (reference)	1.0 (reference)	1.0 (reference)	1.0 (reference)
**Asian**	1.29 (0.96 to 1.73)	1.31 (0.98 to 1.76)	1.41 (1.02 to 1.94)	1.46 (1.06 to 2.01)
**Black**	1.04 (0.68 to 1.58)	1.06 (0.7 to 1.62)	1.42 (0.96 to 2.11)	1.5 (1.01 to 2.23)
**Mixed or other**	1.17 (0.84 to 1.64)	1.18 (0.84 to 1.66)	1.41 (1 to 1.99)	1.49 (1.05 to 2.1)
**Care home resident**	1.39 (1.02 to 1.9) [0.037]	1.4 (1.03 to 1.92) [0.034]	0.56 (0.2 to 1.52) [0.254]	0.57 (0.21 to 1.55) [0.269]
**Comorbidities**	[P<0.001]	[P<0.001]	[0.034]	[0.031]
**None**	1.0 (reference)	1.0 (reference)	1.0 (reference)	1.0 (reference)
**One**	1.78 (1.26 to 2.52)	1.77 (1.26 to 2.5)	1.25 (0.92 to 1.71)	1.25 (0.91 to 1.7)
**Two**	2.03 (1.43 to 2.88)	2.03 (1.43 to 2.87)	1.24 (0.89 to 1.74)	1.24 (0.89 to 1.74)
**Three or more**	2.89 (2.04 to 4.08)	2.89 (2.04 to 4.08)	0.79 (0.54 to 1.15)	0.78 (0.53 to 1.14)
**Pregnant**			0.13 (0.02 to 0.98) [0.048]	0.13 (0.02 to 0.92) [0.042]

**By sample week, London**	[0.273]	[0.22]	[0.007]	[0.013]
16/11/2020	0.34 (0.15 to 0.76)	0.32 (0.14 to 0.72)	0.58 (0.3 to 1.11)	0.59 (0.3 to 1.13)
23/11/2020	0.94 (0.56 to 1.57)	0.89 (0.53 to 1.49)	0.57 (0.31 to 1.03)	0.57 (0.31 to 1.04)
30/11/2020	0.67 (0.4 to 1.12)	0.64 (0.38 to 1.08)	0.33 (0.16 to 0.67)	0.34 (0.17 to 0.7)
07/12/2020	0.95 (0.52 to 1.74)	0.91 (0.5 to 1.67)	0.79 (0.41 to 1.52)	0.82 (0.42 to 1.59)
14/12/2020	0.82 (0.51 to 1.31)	0.81 (0.51 to 1.29)	1.33 (0.85 to 2.08)	1.3 (0.83 to 2.05)
21/12/2020	0.9 (0.61 to 1.33)	0.89 (0.6 to 1.31)	1.04 (0.7 to 1.54)	1.05 (0.7 to 1.57)
28/12/2020	1.0 (reference)	1.0 (reference)	1.0 (reference)	1.0 (reference)
04/01/2021	0.87 (0.51 to 1.51)	0.87 (0.5 to 1.5)	1.11 (0.67 to 1.83)	1.08 (0.65 to 1.8)
**By sample week, Elsewhere**	[0.102]	[0.118]	[0.621]	[0.612]
16/11/2020	1.07 (0.52 to 2.19)	1.06 (0.52 to 2.18)	0.55 (0.1 to 2.91)	0.56 (0.1 to 3.03)
23/11/2020	0.8 (0.38 to 1.67)	0.79 (0.38 to 1.65)	0.56 (0.11 to 2.87)	0.58 (0.11 to 3.02)
30/11/2020	1.14 (0.57 to 2.3)	1.13 (0.56 to 2.28)	0.66 (0.13 to 3.32)	0.7 (0.14 to 3.55)
07/12/2020	0.91 (0.45 to 1.86)	0.88 (0.43 to 1.79)	0.45 (0.08 to 2.61)	0.48 (0.08 to 2.81)
14/12/2020	0.74 (0.37 to 1.47)	0.73 (0.37 to 1.45)	0.38 (0.07 to 2.06)	0.4 (0.07 to 2.2)
21/12/2020	1.01 (0.51 to 2)	0.98 (0.5 to 1.95)	0.81 (0.17 to 3.82)	0.86 (0.18 to 4.09)
28/12/2020	1.27 (0.63 to 2.53)	1.24 (0.62 to 2.48)	1.15 (0.24 to 5.42)	1.22 (0.25 to 5.84)
04/01/2021	1.6 (0.81 to 3.15)	1.54 (0.78 to 3.04)	0.74 (0.15 to 3.55)	0.74 (0.15 to 3.59)

Var., viral variant. P-values are reported from univariate and multivariate Wald tests.

Supplementary Table 2 Hazard ratios for the outcomes of mortality and intensive therapy unit admission associated with lineage B.1.1.7 from mixed effects Cox models, with sensitivity analyses limited to hospital-onset COVID-19 infection (HOCI) and non-HOCI cases.

		**Interaction by sex**	
	**Overall effect of lineage B.1.1.7**	**Effect of lineage B.1.1.7 in males**	**Effect of lineage B.1.1.7 in females**
Mortality			
All inpatients	1.01 (0.79 to 1.28)	0.82 (0.61 to 1.10)	1.30 (0.95 to 1.78)
Excluding HOCI cases	1.2 (0.86 to 1.69)	0.94 (0.63 to 1.4)	1.64 (1.06 to 2.52)
Only HOCI cases	0.83 (0.55 to 1.23)	0.72 (0.43 to 1.19)	0.97 (0.57 to 1.63)
ITU admission			
All inpatients	1.01 (0.75 to 1.37)	0.74 (0.52 to 1.04)	1.82 (1.15 to 2.90)
Excluding HOCI cases	0.88 (0.62 to 1.24)	0.66 (0.45 to 0.97)	1.53 (0.92 to 2.57
Only HOCI cases	0.96 (0.42 to 2.18)	0.74 (0.27 to 2.02)	1.42 (0.42 to 4.77)

Results shown as hazard ratio (95% CI).

Table S3 Proportion of SARS-CoV-2 due to lineage B.1.1.7 for all male inpatient sequenced samples according to patient characteristics

	Lineage B.1.1.7 (n=573)	Not lineage B.1.1.7 (n=610)	Total (n=1183)
**Age Group**			
0-11	12 (75)	4 (25)	16 (100)
12-24	9 (52.9)	8 (47.1)	17 (100)
25-34	27 (65.9)	14 (34.1)	41 (100)
35-49	82 (53.9)	70 (46.1)	152 (100)
50-69	213 (54.1)	181 (45.9)	394 (100)
70-79	107 (41.8)	149 (58.2)	256 (100)
80+	123 (40.1)	184 (59.9)	307 (100)
**Sex**			
Female	0 (NaN)	0 (NaN)	0 (NaN)
Male	573 (48.4)	610 (51.6)	1183 (100)
**Sample week starting:**			
16/11/2020	9 (10.5)	77 (89.5)	86 (100)
23/11/2020	12 (10.3)	105 (89.7)	117 (100)
30/11/2020	31 (24.2)	97 (75.8)	128 (100)
07/12/2020	23 (26.4)	64 (73.6)	87 (100)
14/12/2020	71 (45.5)	85 (54.5)	156 (100)
21/12/2020	117 (57.4)	87 (42.6)	204 (100)
28/12/2020	197 (75.5)	64 (24.5)	261 (100)
04/01/2021	113 (78.5)	31 (21.5)	144 (100)
**Patient Class**			
HCW	1 (33.3)	2 (66.7)	3 (100)
CAI*	439 (55.6)	351 (44.4)	790 (100)
Indeterminate HCAI†	29 (28.7)	72 (71.3)	101 (100)
Probable/definite HCAI‡	104 (36)	185 (64)	289 (100)
**Region**			
Glasgow	40 (30.5)	91 (69.5)	131 (100)
Hampshire	39 (57.4)	29 (42.6)	68 (100)
London	459 (64.3)	255 (35.7)	714 (100)
South Yorkshire	35 (13)	235 (87)	270 (100)
**Ethnicity**			
White	244 (37.7)	403 (62.3)	647 (100)
Black	101 (54)	86 (46)	187 (100)
Asian	62 (72.1)	24 (27.9)	86 (100)
Mixed or other	105 (68.6)	48 (31.4)	153 (100)
Unknown	61 (55.5)	49 (44.5)	110 (100)
**Patient characteristics**			
Obese (BMI>=35)	47 (52.2) [N=573]	43 (47.8) [N=610]	90 (100) [N=1183]
Pregnant	NA	NA	NA
Care home resident	21 (41.2) [N=573]	30 (58.8) [N=609]	51 (100) [N=1182]

**Comorbidities**			
None	181 (58)	131 (42)	312 (100)
One	153 (48)	166 (52)	319 (100)
Two	129 (46.9)	146 (53.1)	275 (100)
Three or more	110 (39.7)	167 (60.3)	277 (100)
Not recorded	0 (NA)	0 (NA)	0 (NA)
**Died within 28d**	112 (38.4) [N=572]	180 (61.2) [N=608]	292 (100) [N=1180]
**Admitted to ITU within 28d⁋**	121 (58.5) [N=554]	86 (41.5) [N=595]	207 (100) [N=1149]

Data shown as *n* (%), with [N] with available data shown where missing values possible. *Diagnosed at or ≤2 days from admission. †Diagnosed 3-7 days from admission. ‡Diagnosed ≥8 days from admission. ⁋Excluding patients admitted to ITU prior to SARS-CoV-2 diagnosis. CAI, community-acquired infection; HCAI, healthcare-associated infection; HCW, healthcare worker; ITU, intensive therapy unit.

Table S4 Proportion of SARS-CoV-2 due to lineage B.1.1.7 for all female inpatient sequenced samples according to patient characteristics

	**Lineage B.1.1.7 (n=534)**	**Not lineage B.1.1.7 (n=624)**	**Total (n=1158)**
**Age Group**			
0-11	3 (37.5)	5 (62.5)	8 (100)
12-24	11 (64.7)	6 (35.3)	17 (100)
25-34	34 (60.7)	22 (39.3)	56 (100)
35-49	77 (58.8)	54 (41.2)	131 (100)
50-69	158 (52.1)	145 (47.9)	303 (100)
70-79	101 (42.6)	136 (57.4)	237 (100)
80+	150 (36.9)	256 (63.1)	406 (100)
**Sex**			
Female	534 (46.1)	624 (53.9)	1158 (100)
Male	0 (NaN)	0 (NaN)	0 (NaN)
**Sample week starting:**			
16/11/2020	6 (6.5)	87 (93.5)	93 (100)
23/11/2020	14 (13.1)	93 (86.9)	107 (100)
30/11/2020	28 (24.1)	88 (75.9)	116 (100)
07/12/2020	32 (27.1)	86 (72.9)	118 (100)
14/12/2020	67 (42.1)	92 (57.9)	159 (100)
21/12/2020	103 (51.8)	96 (48.2)	199 (100)
28/12/2020	164 (74.9)	55 (25.1)	219 (100)
04/01/2021	120 (81.6)	27 (18.4)	147 (100)
**Patient Class**			
HCW	6 (37.5)	10 (62.5)	16 (100)
CAI*	408 (54.7)	338 (45.3)	746 (100)
Indeterminate HCAI†	25 (22.3)	87 (77.7)	112 (100)
Probable/definite HCAI‡	95 (33.5)	189 (66.5)	284 (100)
**Region**			
Glasgow	51 (32.5)	106 (67.5)	157 (100)
Hampshire	35 (63.6)	20 (36.4)	55 (100)
London	412 (67.3)	200 (32.7)	612 (100)
South Yorkshire	36 (10.8)	298 (89.2)	334 (100)
**Ethnicity**			
White	296 (41)	426 (59)	722 (100)
Black	73 (52.5)	66 (47.5)	139 (100)
Asian	56 (55.4)	45 (44.6)	101 (100)
Mixed or other	81 (65.3)	43 (34.7)	124 (100)
Unknown	28 (38.9)	44 (61.1)	72 (100)
**Patient characteristics**			
Obese (BMI>=35)	75 (50.3) [N=534]	74 (49.7) [N=624]	149 (100) [N=1158]
Pregnant	25 (55.6) [N=530]	20 (44.4) [N=624]	45 (100) [N=1154]
Care home resident	24 (32.9) [N=534]	49 (67.1) [N=623]	73 (100) [N=1157]

**Comorbidities**			
None	156 (55.1)	127 (44.9)	283 (100)
One	154 (46.7)	176 (53.3)	330 (100)
Two	132 (44.9)	162 (55.1)	294 (100)
Three or more	92 (36.9)	157 (63.1)	249 (100)
Not recorded	0 (NA)	2 (100)	2 (100)
**Died within 28d**	105 (44.7) [N=534]	130 (55.3) [N=622]	235 (100) [N=1156]
**Admitted to ITU within 28d⁋**	99 (76.2) [N=527]	31 (23.8) [N=619]	130 (100) [N=1146]

Data shown as *n* (%), with [N] with available data shown where missing values possible. *Diagnosed at or ≤2 days from admission. †Diagnosed 3-7 days from admission. ‡Diagnosed ≥8 days from admission. ⁋Excluding patients admitted to ITU prior to SARS-CoV-2 diagnosis. CAI, community-acquired infection; HCAI, healthcare-associated infection; HCW, healthcare worker; ITU, intensive therapy unit.

Figure S1 Bar plot of the proportion of inpatient samples sent for sequencing that sequenced successfully for 6/8 hospitals from which data are available. The proportion of successfully sequenced samples was not different between weeks (chi-squared, p = 0.97).

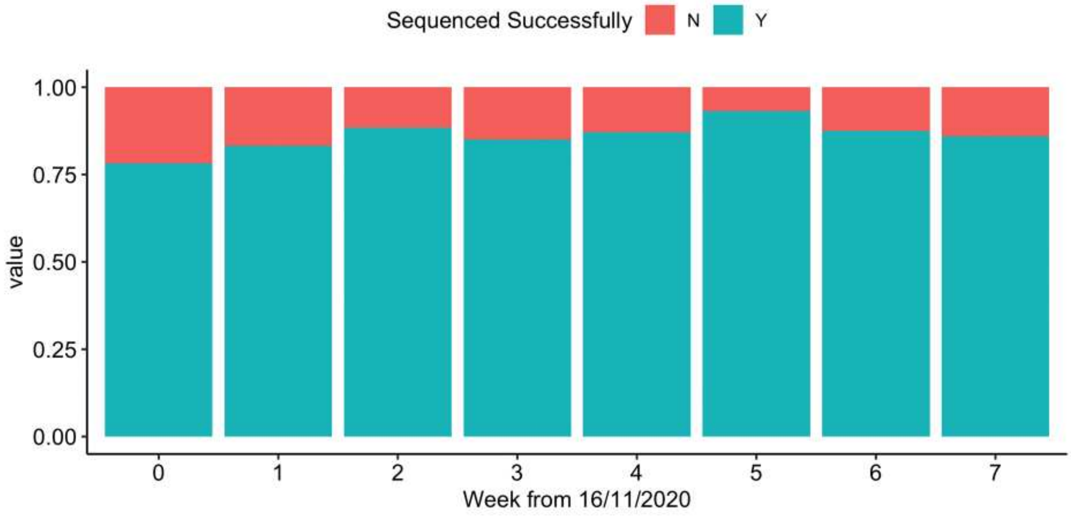

Figure S2 Sequence counts by region, week of sample and patient type.

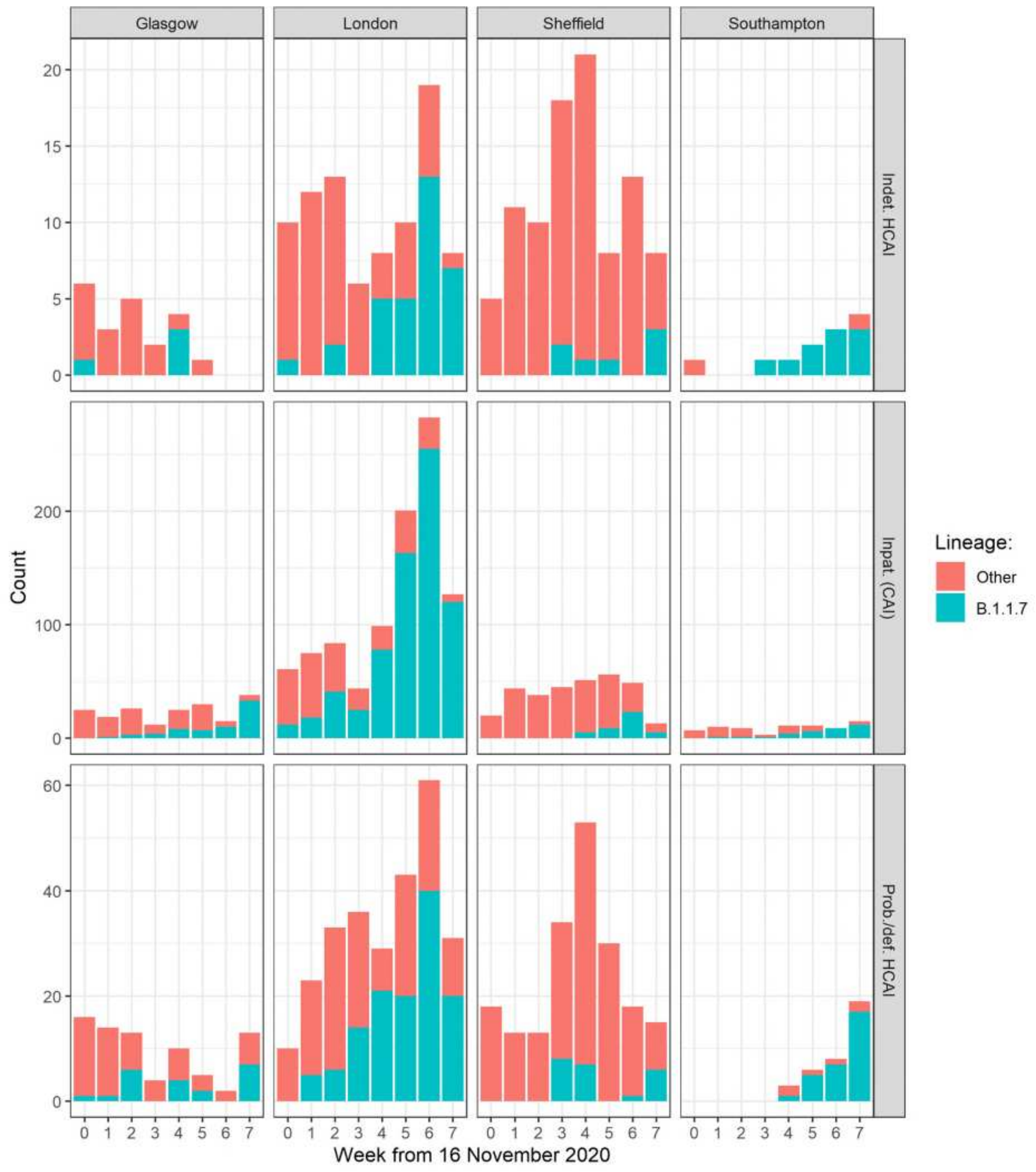

CAI, community-acquired infection; HCAI, healthcare-associated infection.

Figure S3 Kaplan-Meier plots of all-cause mortality among all inpatients admitted with SARS-CoV-2 in relation to lineage B.1.1.7 status, plotted according to patient sex and age categories. Naïve 95%CIs are plotted for illustrative purposes (these are not derived from the multilevel Cox models described).

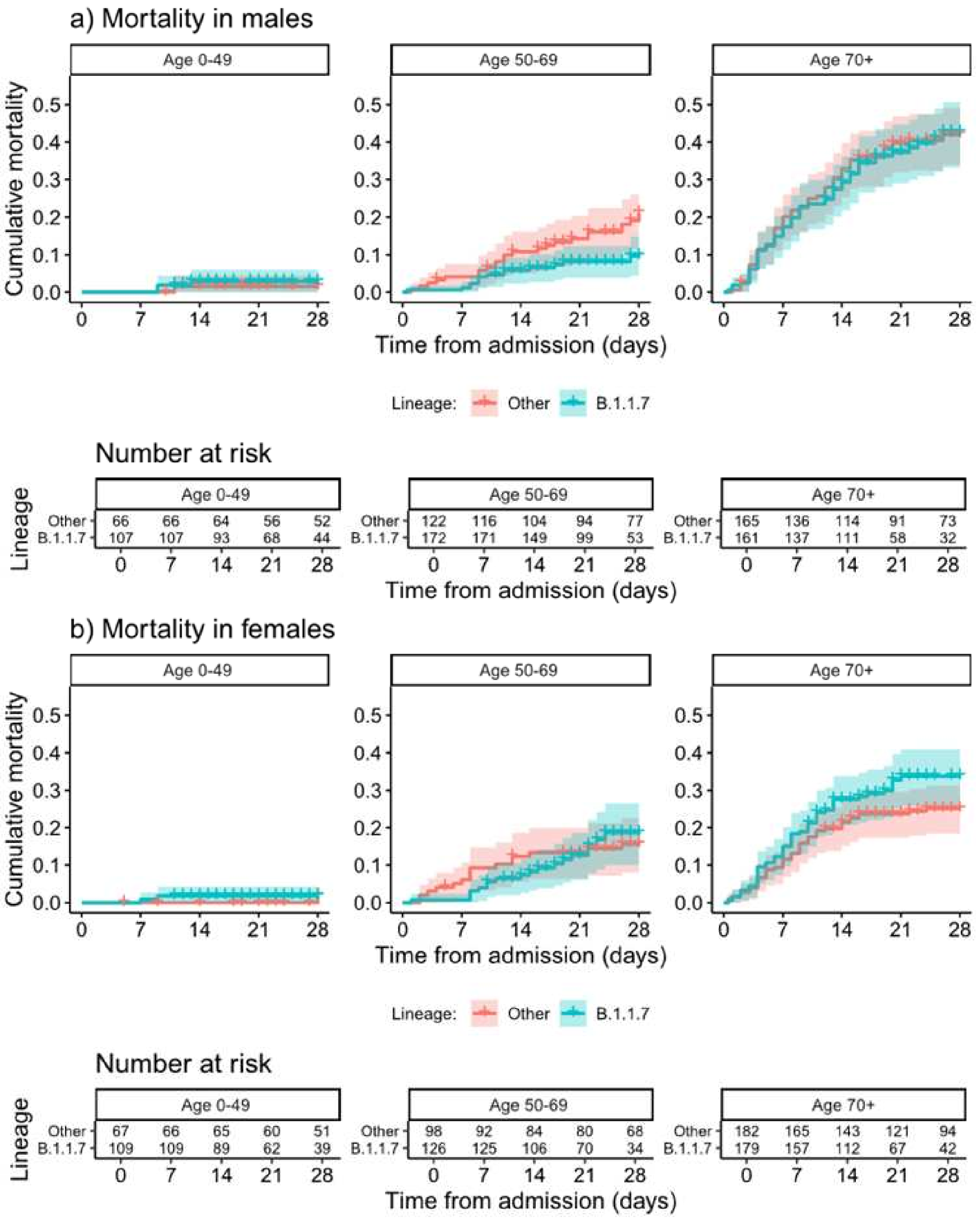

Figure S4 Kaplan-Meier plots of all-cause mortality among all hospital-onset COVID-19 infection cases in relation to lineage B.1.1.7 status, plotted according to patient sex and age categories. Naïve 95%CIs are plotted for illustrative purposes (these are not derived from the multilevel Cox models described).

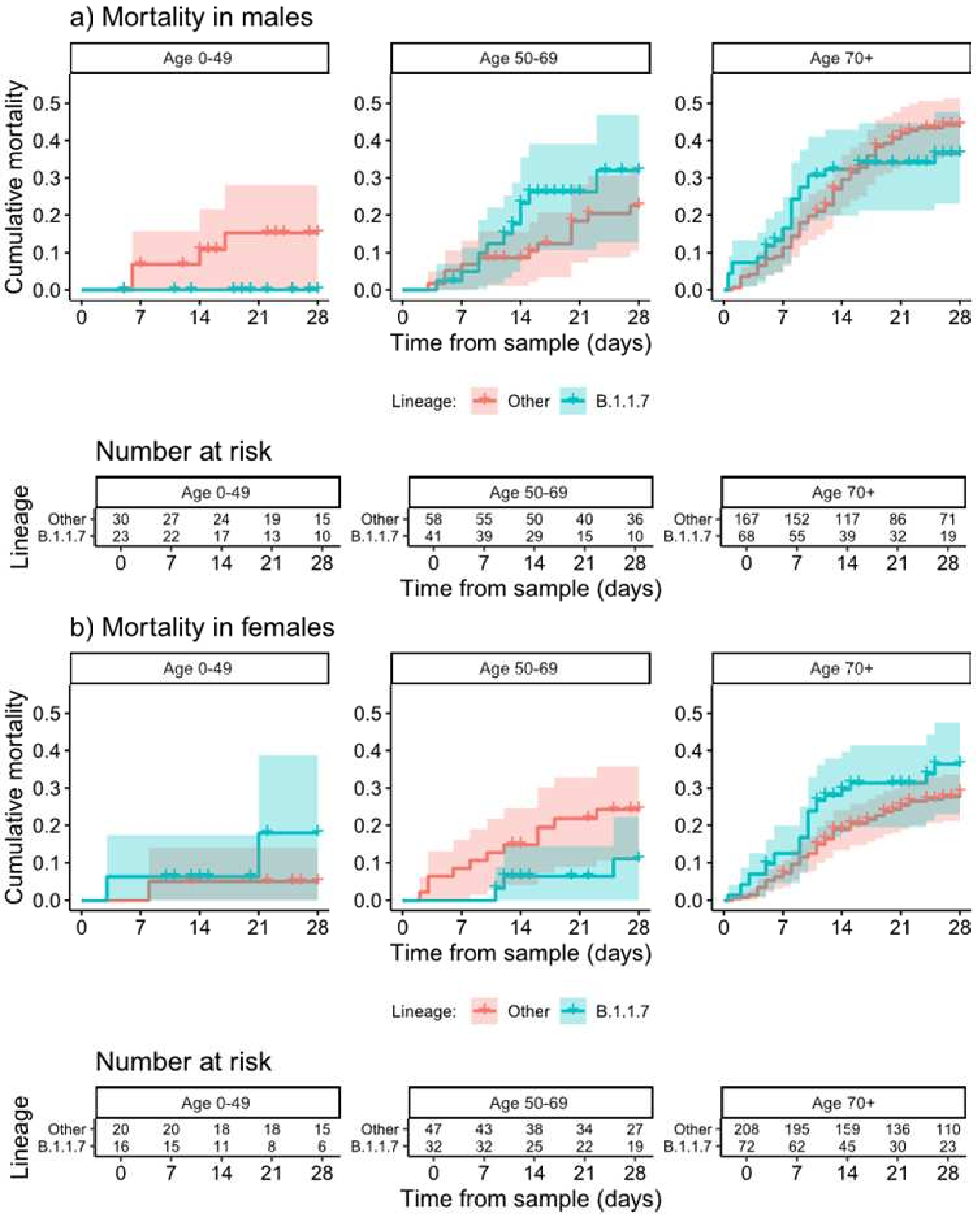

Figure S5 Kaplan-Meier plots of intensive therapy unit (ITU) admission among all inpatients admitted with SARS-CoV-2 in relation to lineage B.1.1.7 status, plotted according to patient sex and age categories. Naïve 95%CIs are plotted for illustrative purposes (these are not derived from the multilevel Cox models described).

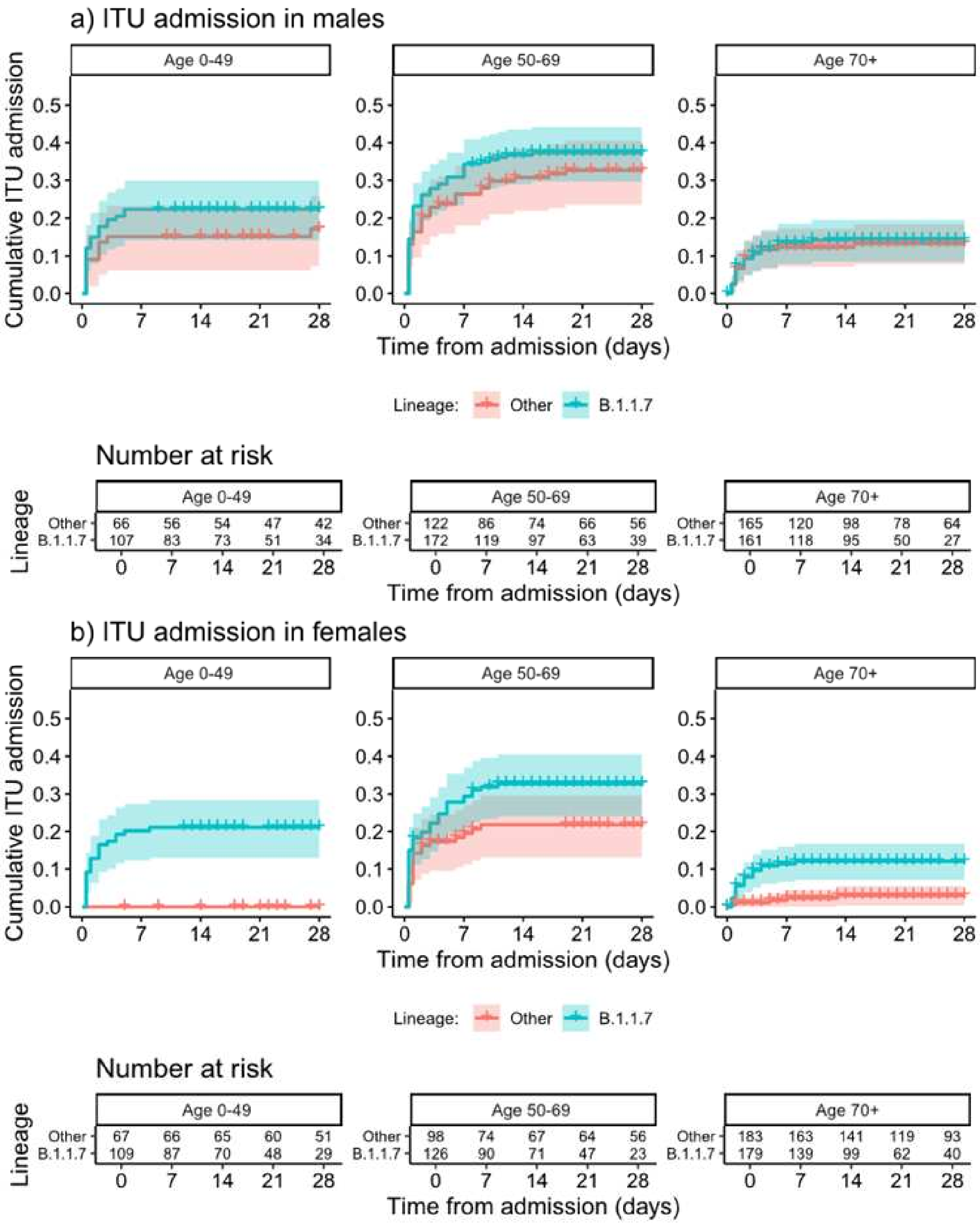

Figure S6 Kaplan-Meier plots of intensive therapy unit (ITU) admission among all hospital-onset COVID-19 infection cases in relation to lineage B.1.1.7 status, plotted according to patient sex and age categories. Naïve 95%CIs are plotted for illustrative purposes (these are not derived from the multilevel Cox models described).

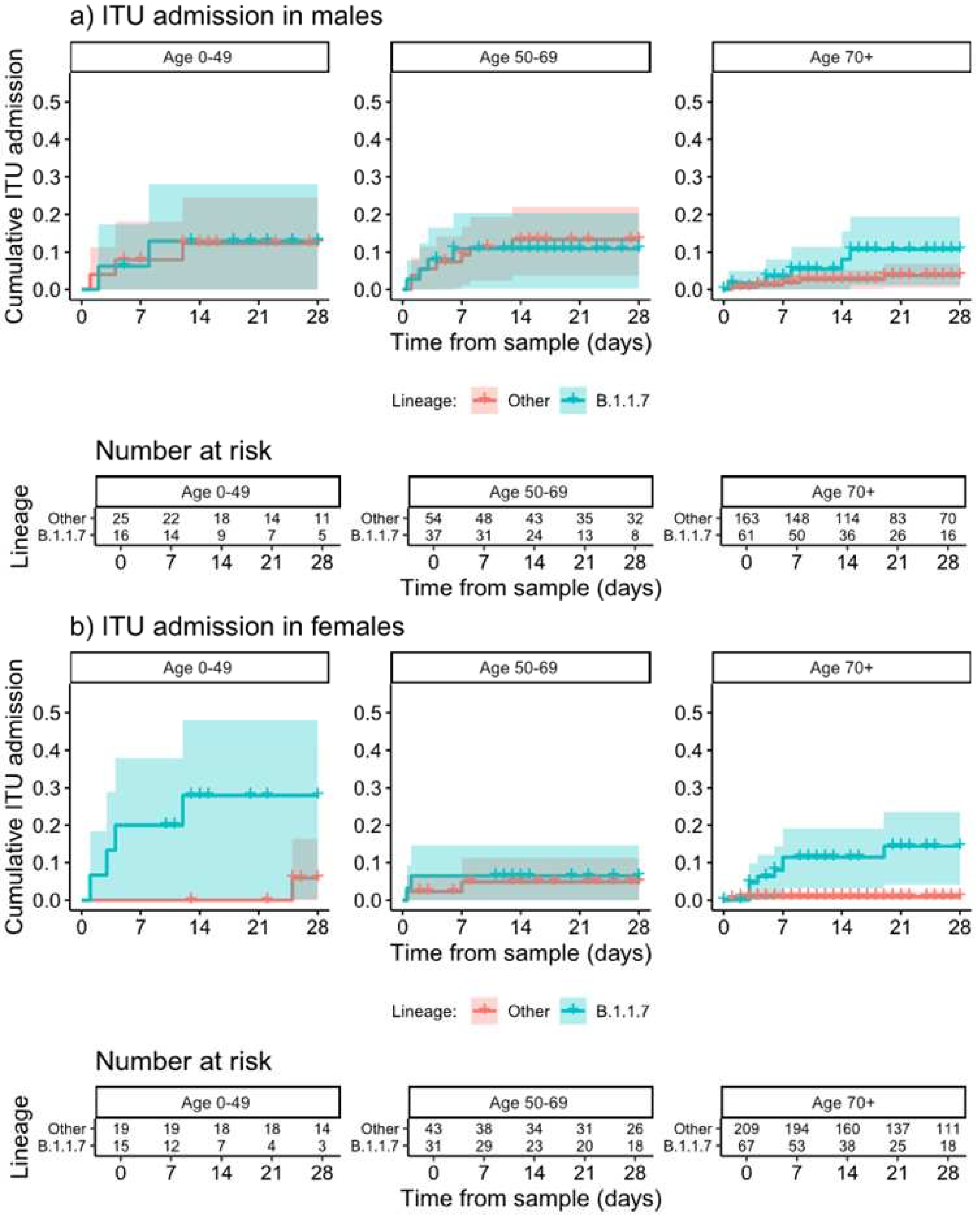

Supplement: Supplementary data [file bmjresp-2021-001029supp001.pdf]
